# *De novo* transcriptome profiling of highly purified human lymphocytes primary cells

**DOI:** 10.1038/sdata.2015.51

**Published:** 2015-09-29

**Authors:** Raoul J.P. Bonnal, Valeria Ranzani, Alberto Arrigoni, Serena Curti, Ilaria Panzeri, Paola Gruarin, Sergio Abrignani, Grazisa Rossetti, Massimiliano Pagani

**Affiliations:** 1 Istituto Nazionale Genetica Molecolare ‘Romeo ed Enrica Invernizzi’, Via F. Sforza 35, Milan 20122, Italy; 2 Department of Clinical Sciences and Community Health, Università degli Studi di Milano, Via Festa del Perdono 7, Milano 20122, Italy; 3 Department of Medical Biotechnology and Translational Medicine, Università degli Studi di Milano, Via Festa del Perdono 7, Milano 20122, Italy

**Keywords:** Long non-coding RNAs, Gene expression analysis, RNA sequencing, CD4-positive T cells

## Abstract

To help better understand the role of long noncoding RNAs in the human immune system, we recently generated a comprehensive RNA-seq data set using 63 RNA samples from 13 subsets of T (CD4^+^ naive, CD4^+^ T_H_1, CD4^+^ T_H_2, CD4^+^ T_H_17, CD4^+^ T_reg_, CD4^+^ T_CM_, CD4^+^ T_EM_, CD8^+^ T_CM_, CD8^+^ T_EM,_ CD8^+^ naive) and B (B naive, B memory, B CD5^+^) lymphocytes. There were five biological replicates for each subset except for CD8^+^ T_CM_ and B CD5^+^ populations that included 4 replicates. RNA-Seq data were generated by an Illumina HiScanSQ sequencer using the TruSeq v3 Cluster kit. 2.192 billion of paired-ends reads, 2×100 bp, were sequenced and after filtering a total of about 1.7 billion reads were mapped. Using different *de novo* transcriptome reconstruction techniques over 500 previously unknown lincRNAs were identified. The current data set could be exploited to drive the functional characterization of lincRNAs, identify novel genes and regulatory networks associated with specific cells subsets of the human immune system.

## Background & Summary

**Figure 1:**
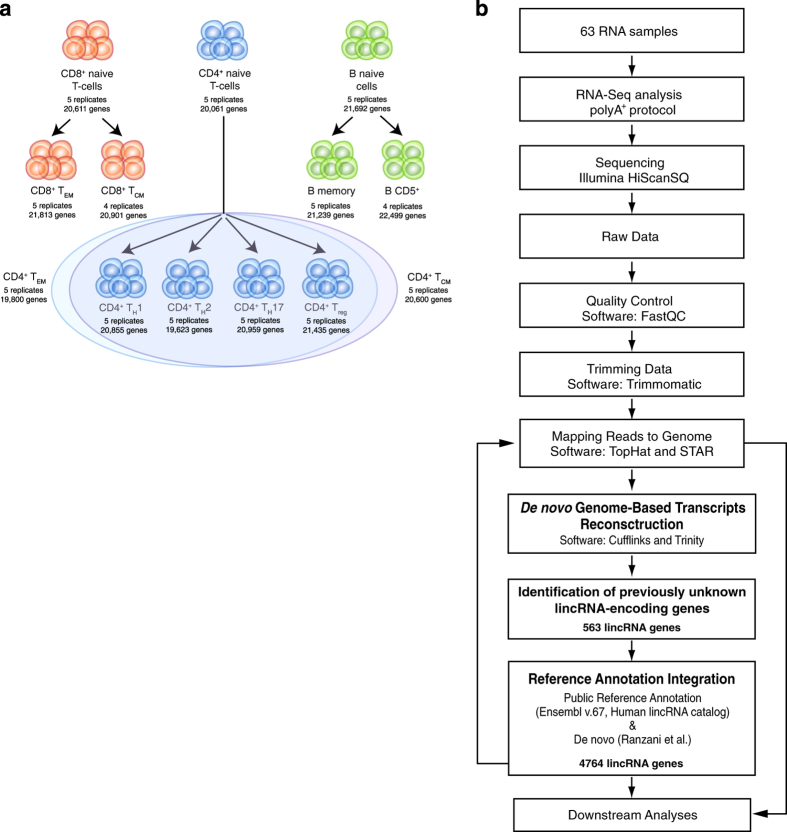
Description of the study: cellular subsets hierarchy and bioinformatics pipeline. (**a**) Hierarchical representation of the different cell subset originating from hematopoietic stem cells. In this study 13 human primary lymphocyte subsets were profiled: CD4^+^ naive; CD4^+^ T_h_1; CD4^+^ T_h_2; CD4^+^ T_h_17; CD4^+^ T_reg_; CD4^+^ T_EM_; CD4^+^ T_CM_; CD8^+^ T_CM_; CD8^+^ T_EM_; CD8^+^ naive; B naive; B memory; B CD5^+^. The number of biological replicates and the expressed genes (FPKM>0.21) for each population is indicated. The total number of samples profiled in this study is 63. (**b**) General overview of the bioinformatic steps and approaches used for the identification of novel lincRNAs.

With emerging technologies it is becoming evident that the vast majority of the genome is transcribed (the so-called ‘dark matter of the genome’) and produces a diverse population of non-protein-coding RNAs (ncRNAs), including long non-coding RNAs (lncRNAs). LncRNAs are transcripts of more than 200 base pairs in length that are often expressed with higher cell-specificity compared to protein-coding genes^1^ despite having lower expression levels. LncRNAs fold in functional domains that allow them to interact with other RNA molecules, DNA and proteins exerting a plethora of different functions in the cells, as chromatin remodeling (XIST, HOTAIR), transcriptional activation or repression (DBE-T, NeSR, lincRNA-Cox2), competition with microRNAs (linc-MD1, PTEN ceRNAs), splicing (sno-lncRNAs), RNA trafficking (NRON), mRNA stability (TINCR), imprinting (KCNQ1OT1) and translation (lncRNA-21p), among others^2^. LncRNAs are also frequently expressed only in specific developmental stages, hinting to their involvement in cell fate determination. Moreover, lncRNAs have been implicated in the maintenance of stem cell pluripotency and differentiation^3^, in the establishment of the cardiovascular lineage and in the control of somatic tissue differentiation^4^. Altogether these findings clearly point out the fundamental role of lncRNAs in the control of cell differentiation and in the maintenance of cell identity. Indeed in the mouse immune system lncRNAs expression changes during naive to memory CD8^+^ T cell differentiation^5^ and during naive CD4^+^ T cells differentiation into distinct helper T cell lineages^6–8^. Our results on human CD4^+^ T lymphocytes specific long intergenic non-coding RNAs (lincRNAs)^7^ are in agreement with the findings in mice. In this work 63 RNA samples from 13 subsets of T (CD4^+^ naive, CD4^+^ T_H_1, CD4^+^ T_H_2, CD4^+^ T_H_17, CD4^+^ T_reg_, CD4^+^ T_CM_, CD4^+^ T_EM_, CD8^+^ T_CM_, CD8^+^ T_EM_, CD8^+^ naive) and B (B naive, B memory, B CD5^+^) lymphocytes were collected. The hierarchy of T and B cells during differentiation of the analyzed subsets is depicted in Fig. 1a as well as the number of biological replicates for each cell population. After RNA-seq sequencing we exploited different *de novo* transcriptome reconstruction approaches that led to the identification of over 500 previously unknown lincRNAs^9^. The general experimental design is shown in Fig. 1b. As recent findings suggest that lncRNAs might contribute to the definition of lymphocytes identity and to the modulation of their functional plasticity, our data set could be used as a resource to guide the validation and functional characterization of lincRNAs and to identify genes and regulatory networks associated with specific cells subsets of the human immune system.

## Methods

### Purification of primary immunological cell subsets

These methods are expanded from our previous article^9^. Blood buffy coat cells of healthy donors were obtained from Fondazione Istituto di Ricovero e Cura a Carattere Scientifico Ca’Granda Ospedale Maggiore Policlinico in Milan, and peripheral blood mononuclear cells were isolated by ficoll-hypaque density-gradient centrifugation. The ethical committee of Fondazione Istituto di Ricovero e Cura a Carattere Scientifico Ca’Granda Ospedale Maggiore Policlinico approved the use of peripheral blood mononuclear cells from healthy donors for research purposes, and informed consent was obtained from subjects. Human blood primary lymphocyte subsets were purified to a purity of >95% by cell sorting through the use of various combinations of surface markers (see Table 1).

### RNA isolation and RNA sequencing

Total RNA was isolated with a mirVana Isolation Kit (Ambion). Libraries for Illumina sequencing were constructed from 100 ng of total RNA with the Illumina TruSeq RNA Sample Preparation Kit v2 (Set A). The libraries generated were loaded on to the cBot automated clonal amplification system (Illumina) for clustering on a HiSeq Flow Cell v3. The libraries clustered on a HiSeq Flow Cell v3 were then sequenced with a HiScanSQ optical imaging system (Illumina). A paired-end run (with a read length of 100 bases) was performed with an SBS Kit v3 DNA sequencing kit (Illumina). Real-time analysis and base calling was performed with HiSeq Control Software (version 1.5, Illumina). CASAVA (version 1.8.2, Illumina) software was used to demultiplex reads into specific sample and groups, the software was configured to operate with ‘--mismatches='1'’ allowing one mismatch during the identification of the indexes (Data Citation 1).

### RNA-seq trimming and mapping

To improve sequence quality, samples data were cleaned by Trimmomatic^10^ (version 0.30) using the following parameters (LEADING:3 TRAILING:3 SLIDINGWINDOW:4:15 MINLEN:36) giving as input the forward and reverse FASTQ sequences for each sample. Only the reads that passed the quality or length threshold on both strands were considered for mapping. The whole data set was aligned to human genome assembly GRCh37 (Genome Reference Consortium Human Build 37) using both TopHat^11^ (version 1.4.1) and STAR^12^ (version 2.2.0). The reference genome was indexed using Bowtie^13^ (version 0.12.9) for TopHat alignment. Both TopHat and STAR were used with default parameters; only for TopHat we specified the mate-inner-dist parameter for each sample of our data set (see the associated Metadata Record). Overall read depth and coverage information of the dataset is reported in Table 2. RNA-seq data from the Illumina Human BodyMap 2.0 project (Data Citation 2) consisting of 16 human tissues were downloaded, processed and mapped using the same criteria.

### Public reference annotation

Ensembl database (version 67 from May 2012, see Data Citation 3) annotation was integrated with a previously published catalogue of lincRNAs^1^ (see Data Citation 4) using Cuffcompare which is provided by the Cufflinks^14^ (version 2.1.1) suite. BioMart was used to categorize Ensembl annotation in different classes by their biotype: ‘lincRNA’ (5,804 genes), protein-coding genes (21,976 genes), receptor-encoding using GO term GO:000487 (2,043 genes encoding molecules with receptor activity function) and the class of genes encoding molecules involved in metabolic processes corresponding to GO term GO:0008152 (7,756 genes). The final public reference annotation consisted of 195,392 transcripts that referred to 62,641 genes, 11,170 of which were non-redundant lincRNA-encoding genes.

### *De novo* genome-based transcripts reconstruction

To identify putative novel genes, not yet annotated and specifically expressed in our datasets, we combined multiple tools and their outputs following a *de novo* genome-based transcripts reconstruction procedure. Samples were aggregated in meta datasets corresponding to the 13 lymphocyte populations. These meta datasets were aligned to the reference genome using two mappers: TopHat and STAR. The resulting 26 alignments were used as independent inputs for Cufflinks configured to use the RABT^15^ assembler for the identification of novel transcripts. The following parameters were used in combination with Cufflinks: ‘-g’ to guide the assembly by the public reference annotation. With these approaches we identified about 3×10^4^ to 5×10^4^ previously unknown transcripts for each lymphocyte population. The third approach was based on the Genome-guided Trinity^16^ pipeline (see Supplementary File 1: example of command lines and Code Availability 9) (release 2012-10-05, http://trinityrnaseq.github.io/#genome_guided) that generates *de novo* transcripts by local assembly on previously mapped reads from specific locations. We used STAR instead of the Trinity’s default aligner GSNAP^17^, as it performed better in terms of both accuracy and computing time. For the first alignment phase STAR was used with the default parameters. The ‘Genome-guided Trinity’ suite was used with the parameters suggested in the main documentation (default). Each candidate transcript was then processed via the Program to Assemble Spliced Alignments^18^ (PASA, http://pasapipeline.github.io/). PASA is a genome annotation tool that reconstructs the complete transcript and gene structures, resolves incongruences derived from transcript misalignments and alternatively splices events, refines the public reference annotation and proposes new transcripts and genes in case no previous annotation can explain the new data. PASA was configured to use STAR as aligner. We recompiled STAR to enable it for handling long reads (putative transcripts); the file ‘IncludeDefine.h’, from the source code, was modified setting the variable ‘MAX_READ_LENGTH’ to a value of ‘100000’. Recompiling the source tree using the GNU ‘make’ utility with the command ‘make STARlong’ generated the desired modified binary version of STAR.

### Identification of previously unknown lincRNA-encoding genes

Data generated by the three different approaches, TopHat/Cufflinks; STAR/Cufflinks; STAR/Trinity, were separately processed to identify unknown lincRNA-encoding genes.

The three *de novo* methods applied to each lymphocyte population, generated transcripts and genes without prior knowledge on their ability to encode for proteins or not. In order to identify only the putative novel lincRNAs, known transcripts and previously unknown isoforms of already annotated genes were filtered out. To perform this filtering we compared the public available reference annotations (see Data Citation 3 and Data Citation 4) with the datasets produced by each approach using a custom script (see Code Availability 10). This comparison can be performed using more consolidated tools as the UCSC bedtools^19^ or Cuffcompare. Transcriptional noise and low polymerase fidelity can create artifactual transcripts therefore only multi-exonic transcripts longer than 200 bases were retained in our analysis. Protein family domains available from Pfam^20^ database (see Data Citation 5) were searched in all transcripts using the HMMER3 (ref. 21) algorithm and those transcripts that matched at least one of all six possible frames were discarded. Another criteria commonly accepted to define lincRNA is the evaluation of their coding potential; absence of coding potential is distinctive of putative lincRNA. PhyloCSF^22^ (cloned from https://github.com/mlin/PhyloCSF on Oct. 2013) is a comparative genomics method (phylogenetic codon substitution frequency) built upon a multiple sequence alignment of 29 mammalian genomes in multi-alignment file format (MAF) (see http://genome.ucsc.edu/FAQ/FAQformat.html#format5 and Data Citation 6). The entire set of novel transcripts that passed the previous filters was used as input for PhyloCSF. Transcripts scoring more than 100 decibans (PhyloCSF scores were obtained using option *--frames=6*) were excluded from the final catalog. This threshold was calculated by Cabili *et al.* ^1^, as it corresponds to a false-negative rate of 6% for coding genes (i.e., 6% of coding genes are classified as noncoding) and a false-positive rate of ~10% (i.e., 9.5% of noncoding transcripts are classified as coding). They optimized PhyloCSF specificity and sensitivity threshold for the classification of coding and noncoding transcripts on the RefSeq reference sequence database of the National Center for Biotechnology Information (RefSeq coding and RefSeq lincRNAs).

### *De novo* transcriptome data integration

In order to create a comprehensive and unique annotation of novel lincRNAs identified in lymphocytes, duplicates generated by the three approaches adopted must be resolved. To accomplish this task Cuffcompare was used. For each *de novo* reconstruction approach Cuffcompare merged the transcripts generated by all the populations. The result is a set of three distinct annotations corresponding to TopHat/Cufflinks, STAR/Cufflinks, STAR/Trinity/PASA. These three lincRNA sets were further merged to generate a non redundant atlas of lincRNAs in human lymphocytes and only those genes identified by at least two out of the three software programs were considered. After data integration through Cuffcompare, a custom script (see Supplementary File 2 and Code Availability 11) was used to remove and substitute the internal gene id (XLOCs) and internal transcript id (TCONs) assigned by the software with their original and public names.

New lincRNAs were then uniquely identified with a name that contains the prefix ‘linc-’; the Ensembl gene name of the nearest protein-coding gene (irrespective of the strand); the location of the lincRNA relative to the sense of transcription of the nearest protein-coding gene: ‘up’ or ‘down’; the description of the concordance of the transcription between the lincRNA and its nearest coding gene: ‘sense’ or ‘antisense’; a counter to distinguish between lincRNA that share the same nearest protein-coding gene. An example of template name is ‘linc-geneX-(up|down)-(sense|antisense)_#n’. The *de novo* annotation has been integrated concatenating it to public reference annotation and the resulting one was used for downstream analyses^7^.

The *de novo* annotation comprises 563 novel lincRNAs genes and 1,797 novel transcripts, published in our previous work^7^ is available in Data Citation 1.

### Code availability

1. CASAVA (version 1.8.2, Illumina, https://support.illumina.com/sequencing/sequencing_software/casava.html), using the mismatch option--mismatches='1'
2. Trimmomatic^10^ (version 0.30, http://www.usadellab.org/cms/index.php?page=trimmomatic), PE, -phred33, LEADING:3, TRAILING:3, SLIDINGWINDOW:4:15, MINLEN:36
3. TopHat^11^ (version 1.4.1, https://ccb.jhu.edu/software/tophat/index.shtml), mate-inner-dist was set for each dataset to the InnerSize filed available from Supplementary Table 1
4. STAR^12^ (version 2.20, https://github.com/alexdobin/STAR/releases), default
5. Bowtie^13^ (version 0.12.9, http://bowtie-bio.sourceforge.net/index.shtml), default
6. Cufflinks^14^ (version 2.1.1, http://cole-trapnell-lab.github.io/cufflinks/), option: -g Tells Cufflinks to use the supplied reference annotation a GTF file to guide RABT assembly.
7. HMMER3 (ref. 21) (version 3.0, http://hmmer.janelia.org/), default
8. PhyloCSF^22^ (release Oct. 2013, https://github.com/mlin/PhyloCSF), --frames=6
9. Custom template for Trinity/Pasa, Supplementary File 1, https://gist.github.com/helios/7cb7a6afbe625749e824 under GPL v3.0
10. Custom Biogem package, freely available at https://github.com/ingm-oss/bioruby-genomic-intervals under GPL v3.0
11. Custom rename_gtf.rb, Supplementary File 2, a ruby script freely available at https://github.com/ingm-oss/rename_gtf under GPL v3.0

## Data Records

In this study we deposited 1 dataset, which contains the RNA-Seq raw reads in FASTQ format (see Data Citation 1 and Supplementary Table 1),which is a simplified version of the ISA-TAB (see the associated Metadata Record). This dataset contains 63 samples in total, grouped by 13 lymphocyte subsets with 4 or 5 biological replicates each. Supplementary Table 1 is an XLSX with the following header: Source, the original source name used by the lab; Name, assigned by the provider; SubSet, the lymphocyte subset; Antibody, the antibodies used for sorting; InnerSize, the estimated inner size; R1 URI, the forward reads uri for download; R1 MD5SUM, checksum for the forward reads; R2 URI, the reverse reads uri for download; R2 MD5SUM, checksum for the reverse reads. The annotation of the 563 newly described lincRNA (see Data Citation 1: the new 563 annotated lincRNAs^7^) is a General Transfer Format (GTF).

## Technical Validation

### RNA-seq raw data quality

**Figure 2:**
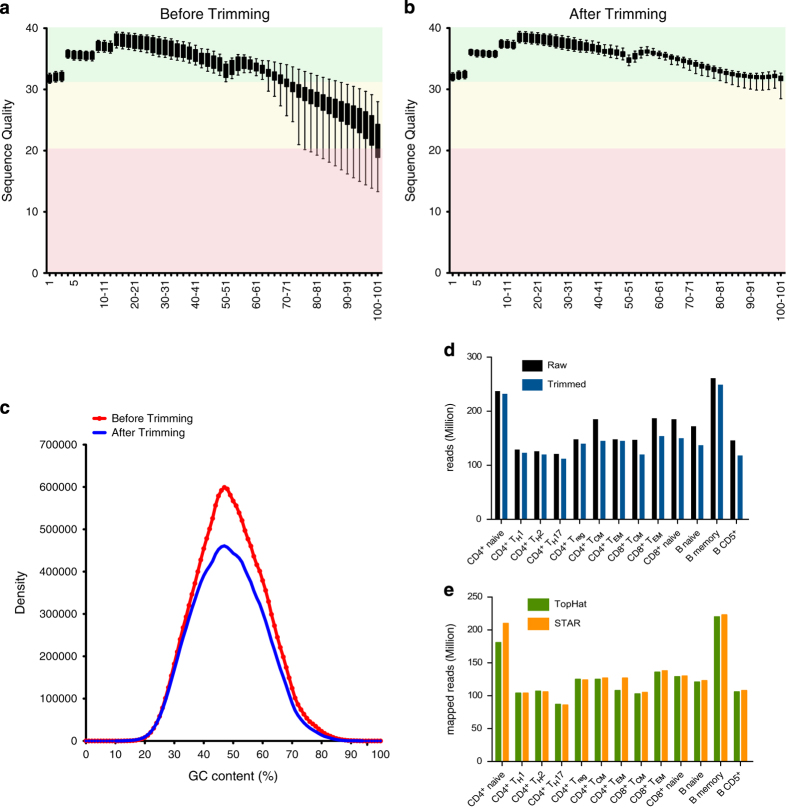
Quality control assessments. (**a**) Phred quality score of the average distribution over all reads across all samples in each base before and (**b**) after trimming. (**c**) %GC content before and after trimming. (**d**) Detailed overview of the human lymphocyte subsets profiled: raw reads (black), the reads trimmed and filtered by quality (blue), and (**e**) the comparison of the mapped reads using TopHat (light green) and STAR (light orange).

Assessing the quality of the data performing the Quality Control (QC) is crucial to the whole study. RNA-seq data generated were initially analyzed with FastQC and a summary plot with the data from all samples is depicted in Fig. 2a. The quality of the reads during the sequencing tends to decrease but it can be further improved using specific software that removes low quality bases reducing the length of the read or directly discard the whole read when its quality is too low. To perform the trimming and filtering Trimmomatic was run on each sample and the data were later on reanalysed with FastQC to confirm the quality improvements. The summary of the resulting data is shown in Fig. 2b Another criteria to measure the QC for NGS reads is the % of GC content, which is improved by the filtering (Fig. 2c). Moreover the trimming step did not dramatically decrease the final number of reads (Fig. 2d).

During the study two mapping software were used, TopHat and STAR. To exclude the possibility of discordance between the two aligners, the mapping results were compared to assess their mapping performance. The alignments with the two software showed a good concordance (96%) with a slight advantage of STAR in terms of mapped reads (Fig. 2e).

### RNA-seq biological replicates

**Figure 3:**
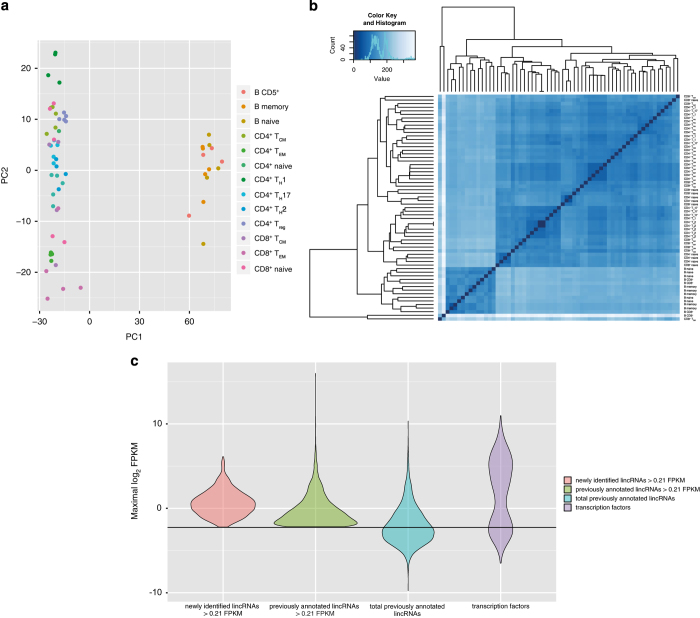
Analysis of intra-population consistency: Principal Component Analysis and hierarchical clustering. (**a**) Principal Component Analysis (PCA) performed using DESeq2 rlog-normalized RNA-seq data. Loadings for principal components 1 (PC1) and PC2 are reported in graph (on x and y-axes). (**b**) Hierarchical clustering analyses performed using DESeq2 rlog-normalized RNA-seq data. Color code (from white to dark blue) refers to the distance metric used for clustering (dark blue corresponds to the maximum of correlation values). (**c**) Violin plot of the normalized FPKM values for the newly identified lincRNAs, previously annotated lincRNAs and transcription factors genes. The black line represents the normalized FPKM threshold (0.21 FPKM).

Biological replicates are fundamental to guarantee data consistency, in this study the lymphocyte populations profiled have 4 biological replicates for B CD5^+^ and CD8^+^ T_CM_ and 5 biological replicates for all the other populations. In order to establish the congruency among biological replicates Principal Component Analysis (PCA) (Fig. 3a) and hierarchical clustering (Fig. 3b) were performed. A good separation between B and T cells samples is achieved by PCA on normalized read counts using DESeq2 (ref. 23). Comparable results are obtained using hierarchical clustering on the same data. Moreover, similarity between biological replicates of the same population showed a good consistency and correlation among them.

### *De novo* transcripts identification

Multiple combinations of software and filters were used for the identification of lincRNAs in the 13 lymphocytes populations. Moreover, we considered only newly described lincRNAs detected in at least 2 out of 3 *de novo* approaches to improve the reliability of the data.

LincRNAs discrimination between coding and non-coding RNA depends on the algorithm used to asses the coding potential, in this study was used PhyloCSF. The final dataset of putative lincRNAs was further processed using iSeeRNA^24^ (webserver version 1.2.2) in order to verify our results using a different approach based on Support Vector Machines (SVM). The classification we obtained is highly concordant, in fact ~99% of the putative lincRNAs contained in the final catalogue (see Data Citation 1) are classified as 'noncoding' also by iSeeRNA.

### Expression threshold definition

As reported in literature, many lincRNAs are expressed at lower levels than protein coding genes^25^, so definition of a FPKM threshold would contribute to discriminate low abundant functional transcripts from technical or biological noise. In a recent study^26^ an approach based on the integration of RNA-seq and CHIP-seq data was used for the definition of a sensible FPKM threshold. 17 human cell lines from ENCODE project were analyzed to establish a relationship between gene expression levels and promoters activities. The expression cutoff was set where the fraction of genes associated to active promoters is equal to the fraction of them associated to repressed promoters. We considered a threshold of 0.21 FPKM that is the mean of the data reported for different cell types in the paper. In Fig. 3c is shown that newly identified lincRNAs (selected with FPKM expression values >0.21), have higher expression levels compared to previously annotated ones with expression values above the threshold and with no threshold (light green and light blue). We then considered for the downstream analysis only genes whose expression values were at least 0.21 FPKM in one population.

## Usage Notes

This study was performed on the version 67 from May 2012 of Ensembl GRCh37. In order to access and use the catalogue of newly described lincRNAs generated in this study (see Data Citation 1 ), researchers must update it to the most updated genome version using the liftover software from UCSC (https://genome.ucsc.edu/cgi-bin/hgLiftOver) or the assembly converter from Ensembl (http://www.ensembl.org/Homo_sapiens/Tools/AssemblyConverter).

Software used during this study went through minor and major code base updates. The more notable software suite that has been updated during the time is Trinity and is strongly suggested to use the latest release downloadable from https://github.com/trinityrnaseq/trinityrnaseq.

For the evaluation of the coding potential of *de novo* transcripts we suggest to use other recently developed software that perform the classification more efficiently than PhyloCSF, such as iSeeRNA, CNCI^27^ and CPAT^28^. It has been demonstrated that these algorithms have a higher level of accuracy, and execution times are considerably faster.

Our transcriptome analysis includes thirteen human primary T and B cells whereas most of the available immune system datasets are limited to mice samples, cell lines or *in vitro* expanded cells. Therefore this study represents a valuable resource for those researches who need to access and analyze the expression patterns of both coding and non-coding transcripts in human lymphocytes. Moreover the thorough analysis we performed to assess the expression of both novel and previously annotated lincRNAs in these cells sets the grounds for further studies on the still largely uncharacterized function of long non-coding RNA in human lymphocytes subsets.

## Additional Information

**How to cite this article:** Bonnal, R. J. P. *et al.* *De novo* transcriptome profiling of highly purified human lymphocytes primary cells. *Sci. Data* 2:150051 doi: 10.1038/sdata.2015.51 (2015).

## Supplementary Material



Supplementary Table 1

Supplementary File 1

Supplementary File 2

## Figures and Tables

**Table 1:** Purification and RNA-Seq of human primary lymphocyte subsets

**Subset**	**Purity (%)**	**Sorting phenotype**	**Donors**
CD4^+^ naive	99,8±0,1	CD4^+^ CCR7^+^ CD45RA^+^ CD45RO^−^	5
CD4^+^ T_H_1	99,9±0,05	CD4^+^ CXCR3^+^	5
CD4^+^ T_H_2	99,7±0,3	CD4^+^ CRTH2^+^ CXCR3^−^	5
CD4^+^ T_H_17	99,1±1	CD4^+^ CCR6^+^ CD161^+^ CXCR3^−^	5
CD4^+^ T_reg_	99,0±0,8	CD4^+^ CD127^−^ CD25^+^	5
CD4^+^ T_CM_	98,4±2,8	CD4^+^ CCR7^+^ CD45RA^−^ CD45RO^+^	5
CD4^+^ T_EM_	95,4±5,5	CD4^+^ CCR7^−^ CD45RA^−^ CD45RO^+^	5
CD8^+^ T_CM_	98,3±0,8	CD8^+^ CCR7^+^ CD45RA^−^ CD45RO^+^	4
CD8^+^ T_EM_	96,8±0,9	CD8^+^ CCR7^−^ CD45RA^−^ CD45RO^+^	5
CD8^+^ naive	99,3±0,2	CD8^+^ CCR7^+^ CD45RA^+^ CD45RO^−^	5
B naive	99,9±0,1	CD19^+^ CD5^−^ CD27^−^	5
B memory	99,1±0,8	CD19^+^ CD5^−^ CD27^+^	5
B CD5^+^	99,1±0,8	CD19^+^ CD5^+^	4
Purity achieved (middle left) by the sorting of 13 human lymphocyte subsets (isolated from peripheral blood lymphocytes of four to five different donors per subset) by various surface marker combinations (Sorting phenotype). T_reg_,regulatory T cells; T_CM_, central memory T cells; T_EM_, effector memory T cells; B, B cells. Data are representative of at least four experiments (mean±s.d. for purity).			

**Table 2:** Overall read depth and coverage information

**Subset**	**Raw**	**Trimmed**	**TopHat**	**STAR**
CD4^+^ naive	237	232	185	210
CD4^+^ T_H_1	129	123	104	104
CD4^+^ T_H_2	126	120	107	106
CD4^+^ T_H_17	121	112	87	86
CD4^+^ T_reg_	148	140	125	124
CD4^+^ T_CM_	185	145	125	127
CD4^+^ T_EM_	148	145	151	127
CD8^+^ T_CM_	147	120	103	105
CD8^+^ T_EM_	187	154	136	138
CD8^+^ naive	185	150	129	130
B naive	172	137	121	123
B memory	261	249	220	223
B CD5^+^	146	118	106	108
Data aggregated by population, the number of raw reads, the number of trimmed reads and the number of mapped reads for both TopHat and STAR on the Ensembl human sequence, version 67 from May 2012. Number of raw reads, trimmed reads and mapped reads for both TopHat and Star are reported for all 13 lymphocytes populations.				
